# Role of Chitinase 3-Like 1 Protein in the Pathogenesis of Hepatic Insulin Resistance in Nonalcoholic Fatty Liver Disease

**DOI:** 10.3390/cells10020201

**Published:** 2021-01-20

**Authors:** Songhua Zhang, Aryanna Sousa, Mengqui Lin, Ayako Iwano, Rishubh Jain, Bing Ma, Chang Min Lee, Jin Wook Park, Suchitra Kamle, Rolf Carlson, Ghun Geun Lee, Jack A. Elias, Jack R. Wands

**Affiliations:** 1 Liver Research Center, Rhode Island Hospital and the Warren Alpert Medical School of Brown University, Providence, RI 02903, USA; songhua_zhang@brown.edu (S.Z.); aryanna.sousa@gmail.com (A.S.); mengqi_lin@alumni.brown.edu (M.L.); ayako.iwano23@gmail.com (A.I.); rishubh_jain@brown.edu (R.J.); rcarlson@lifespan.org (R.C.); 2 Department of Molecular Microbiology and Immunology, Brown University, Providence, RI 02912, USA; bing_ma@brown.edu (B.M.); chang-min_lee@brown.edu (C.M.L.); jinwoook_park@brown.edu (J.W.P.); suchitrakamle@gmail.com (S.K.); chun_lee@brown.edu (G.G.L.); jack_elias@brown.edu (J.A.E.); 3 Department of Medicine, Warren Alpert Medical School of Brown University, Providence, RI 02912, USA

**Keywords:** nonalcoholic steatosis (NASH), Chi3L1, hepatic insulin resistance, anti-Chi3L1 monoclonal antibody

## Abstract

A recently discovered human glycoprotein, chitinase 3-like 1 (Chi3L1), may play a role in inflammation, tissue remodeling, and visceral fat accumulation. We hypothesize that Chi3L1 gene expression is important in the development of hepatic insulin resistance characterized by the generation of pAKT, pGSK, and pERK in wild type and Chi3L1 knockout (KO) murine liver following insulin stimulation. The Chi3L1 gene and protein expression was evaluated by Real Time PCR and ELISA; lipid accumulation in hepatocytes was also assessed. To alter Chi3L1 function, three different anti-Chi3L1 monoclonal antibodies (mAbs) were administered in vivo and effects on the insulin signaling cascade and hepatic lipid deposition were determined. Transmission of the hepatic insulin signal was substantially improved following KO of the CHi3L1 gene and there was reduced lipid deposition produced by a HFD. The HFD-fed mice exhibited increased Chi3L1 expression in the liver and there was impaired insulin signal transduction. All three anti-Chi3L1 mAbs partially restored hepatic insulin sensitivity which was associated with reduced lipid accumulation in hepatocytes as well. A KO of the Chi3L1 gene reduced lipid accumulation and improved insulin signaling. Therefore, Chi3L1 gene upregulation may be an important factor in the generation of NAFLD/NASH phenotype.

## 1. Introduction

Nonalcoholic fatty liver disease (NAFLD) is the most common hepatic disorder in the world today and affects approximately 25% of adults in the United States. Approximately 20% of NAFLD patients may gradually progress to the more severe nonalcoholic steatohepatitis (NASH) [1,2]. The NASH phenotype is pathologically characterized by inflammation, hepatocyte injury, lipid degeneration and fibrosis, which can advance to cirrhosis and hepatocellular carcinoma (HCC) over time [1]. If untreated, NASH may become one of the most frequent and serious causes of liver failure, cirrhosis and the need for liver transplantation in the near future [1,3].

There are many rodent models of nonalcoholic fatty liver disease [4]. However, some that produce insulin resistance will include ethanol in the feeding regimen thereby promoting hepatic damage and intense steatosis [5,6]. This is an important consideration since dietary ethanol consumption produces insulin resistance in the liver of rodent models [7,8,9]. Therefore, we have not included ethanol as a dietary component in these studies. We chose to feed mice with the B6 background a high fat diet for 16 weeks which has been shown to produce panlobular steatosis, inflammation and induced fibrosis as previously described [10].

A recently discovered molecule called chitinase 3 like protein 1 (Chi3L1) is a member of the 18 glycosyl hydrolase gene family, which can bind to chitin with no enzymatic activity [11]. Alternative names for Chi3L1 are YKL-40 in humans and breast regression protein-39 in rodents. Chitinase-like proteins may serve as biomarkers for human diseases [11,12]. Previous studies have shown that Chi3L1 plays an important role in inflammation, tissue remodeling and cancer. It is now known to be expressed by a variety of cells including macrophages, neutrophils, epithelial cells and perhaps hepatocytes and is stimulated by a number of mediators including IL-13, IL-6, IL1β, and IFN-γ [12,13,14]. The Chi3L1 stimulates Th2 inflammation and M2 macrophage differentiation, inhibits oxidant injury, controls inflammasome and caspase activation, regulates TGF-β1 elaboration, contributes to antibacterial responses and activates MAP kinase (MAPK), Akt/protein kinase B and Wnt/β-catenin signaling [14,15,16]. Many of these responses are mediated by a multimeric receptor called chitosome that contains an IL-13Rα2 and a TMEM219 (TMEM) β subunit [16,17]. In keeping with these diverse sources and stimuli, elevated levels of Chi3L1 have been noted in a wide variety of diseases characterized by inflammation, fibrosis and tissue remodeling [12,13,14,18]. Its role in inflammatory liver disease such as NAFLD/NASH has not been determined. However, it appears to regulate visceral fat accumulation and asthma-like Th2 inflammation [12,19,20,21]. Recent clinical studies suggest that Chi3L1 serum levels may be related to different stages of liver fibrosis [22,23]. There is a preliminary report to suggest that when compared with established biomarker scoring systems, Chi3L1 gene expression may be used to distinguish between isolated simple steatosis vs. NASH by whole genome RNA sequencing (RNA-seq) [24]. More recently, investigators revealed that increased Chi3L1 expression levels were involved in the progression of NASH in a murine model [25]. However, there is little information on how enhanced Chi3L1 expression contributes to NASH pathogenesis. The development of NASH is strongly associated with obesity, insulin resistance and the metabolic syndrome [1,26,27]. In this context, serum levels of Chi3L1 may be linked to insulin resistance and obesity [21,28,29]. We provide evidence on how Chi3L1 protein expression is linked to components of the insulin cascade and alters signaling in the intact murine liver during the development of steatohepatitis [7,30] and how this effect can be partially reversed by parenteral anti-Chi3L1 mAb treatment.

## 2. Materials and Methods

### 2.1. Animal Studies

Protocols for Chi3L1 KO mice and C57BL/6 WT mice used in this study were approved by the Institutional Animal Care and Use Committee (IACUC) at Brown University. Experimental procedures were conducted according to the guidelines of the Institutional Animal Care and Use Committee. The diets were comprised of standard chow and a high fat diet (HFD) [19] and administered over several weeks. The following approaches were used in this study:

(1) Diet: Chow diet (CD) and high fat diet (HFD) were purchased from ENVIGO (Cat# TD88137; Indianapolis, IN, USA). The HFD includes 60% fat, 20% carbohydrate which produce striking hepatic lipid accumulation when given for 16 to 20 weeks; there is also evidence of hepatocyte injury, inflammation, steatosis, and insulin resistance under these experimental conditions [31]. (2) Knockout mice (Chi3L1^−/−^): Breast regression protein 39 (BRP-39)/Chi3L1 KO mice were generated, characterized and validated as described [14]. After breeding for more than 10 generations onto C57BL/6 mice, these Chi3L1^−/−^ mice were similar to C57BL/6 littermates in size, rate of growth, physical activity levels [14]. Six to eight-week old Chi3L1 KO mice weighing approximately 20 g were used for this study. Three Chi3L1 KO mice and three C57BL/6 WT mice were fed with CD and euthanized in one month to test the hypothesis that Chi3L1 may play a role in generation of hepatic insulin resistance. In order to evaluate if HFD can produce fat accumulation in Chi3L1 knockout mice, we fed them with this HFD diet for 16 weeks and compared lipid deposition levels in the liver. The WT mice fed CD served as an experimental control group. (3) C57BL/6 wild type (WT) mice: In order to study if anti-Chi3L1 mAbs can improve hepatic insulin sensitivity by altering its function, ten WT mice fed with HFD were divided into two groups of 5 each. All were fed HFD and treated with either a nonrelated IgG mAb or one of the anti-Chi3L1 mAb (FRG) for 12 weeks at 200 μg/mouse, given twice per week.

In parallel, and to confirm results, we employed five groups of WT mice (N = 40) to study the effects of additional Chi3L1 mAb administration on HFD induced lipid accumulation in the liver. The experiment contained one control group (N = 8) in which mice were fed with standard CD, while animals in the other four experimental groups were fed HFD for 16 weeks. As shown in Table 1, group 1 & 2 were untreated versus the other groups administered different anti-Chi3L1 mAbs in groups 3, 4, and 5. Mice in group 3 were treated with an unrelated mAb IgG (200 μg/mouse) twice a week i.p. Group 4 and 5 were given different anti-Chi3L1 mAb antibodies (200 μg/mouse) twice a week.

### 2.2. Human Liver Biopsy Specimens

We collected twenty archival liver specimens from ten patients with NAFLD (N = 5) and NASH (N = 5) who underwent percutaneous liver biopsies for clinical diagnosis. This study involving IHC staining for Chi3L1 expression was approved by the Rhode Island Hospital institutional review board (IRB).

### 2.3. Preparation of Monoclonal Anti-Chi3L1 Antibodies

To further clarify the effects of Chi3L1 protein expression on hepatic insulin resistance and hepatosteatosis, we generated three monoclonal anti-Chi3L1 antibodies in an attempt to alter its function as follows: (1) The murine Chi3L1 mAb (FRG) was generated using FRG peptide antigen (QEDASPDRF, amino acid 223–234 of human Chi3L1) as the immunogen. This mAb specifically detects both human and mouse Chi3L1 with high affinity (kd ≈ 1 × 10^−9^) and has neutralizing activities. (2) In addition, two other such antibodies of high affinity and high specificity were produced using established techniques [32]. In brief, recombinant Chi3L1 protein was employed as an antigen to immunize Balb/c mice as described [32]. Two anti-Chi3L1 mAbs (CH568 and CHXI3B6) were selected, cloned and purified (Supplemental Figure S1). These reagents were highly immunoreactive against Chi3L1 protein by ELISA binding assays (data not shown and available upon request) and used in treatment groups 4 and 5 (Table 1).

### 2.4. Insulin Stimulation

In vivo insulin stimulation of the hepatic signaling cascade was performed as previously described [33]. Time course experiments (Supplemental Figure S2) established that fifteen minutes after intraperitoneal (i.p.) insulin administration (1 Unit/gram body weight) to the murine liver, phosphoprotein kinase B (PKB or pAKT) expression reached a peak value. Since pAKT activation at the carboxyl terminus serine (Ser 473) plays a key role in the upstream events of the insulin signaling cascade, all western blots generated were obtained 15 min after i.p. insulin stimulation. In groups 1 to 5, four of the mice in each group received insulin stimulation while the other four remained unstimulated and served as controls.

### 2.5. Measurement of Chi3L1 Gene Expression

Total RNA was extracted from murine liver tissue using Trizol reagent (Invitrogen, Carlsbad, CA, USA), and then purified with RNeasy mini-spin column (Qiagen, Germantown, MD, USA). Total RNA (1 μg) of each liver sample was applied for cDNA synthesis with iScript™ kit (Bio-Rad Laboratories, Hercules, CA, USA) according to the manufacturer’s protocol.

Quantitative real time polymerase chain reaction (PCR) was used to evaluate Chi3L1 gene expression in the mouse liver. Synthesized cDNA from the prior step was used as a template for real time PCR, which was carried out with Chi3L1 gene specific primer set, a QuantiTect^®^ SYBR^®^ Green PCR kit (Qiagen) and performed on a StepOnePlus Real-Time PCR System (Applied Biosystems, Foster City, CA, USA). The cycling condition was as follows: 95 °C for 15 min, then 40 cycles of 94 °C for 15 s, 60 °C for 30 s and 72 °C for 30 s. Reference gene glyceraldehyde 3-phosphate dehydrogenase (GAPDH) was used as an internal control to normalize quantitative gene expression. The relative mRNA expression levels were analyzed with the 2^−ΔΔCT^ method. The following primer sets were used in this study. Mouse Chi3L1 gene, forward TGGATCTCGCCTGGCTCTAC, Reverse CTGCGCTGAGCAGGAGTTTC. Housekeeping gene GAPDH, forward AGGGCTGCTT TTAACTCTGGT; Reverse CCCCACTTGATTTTGGAGGGA [34,35].

### 2.6. Protein Extraction

Proteins were extracted by homogenizing a mouse liver section with ice-cold tissue lysis buffer. The supernatant was collected after centrifugation and the lipid on the surface of the supernatant was removed. Protein concentration was measured by bicinchoninic acid assay (BCA assay, Pierce, Thermo Fisher Scientific, Waltham, MA, USA). In addition, a slice of liver tissue was fixed with 10% formalin for morphologic analysis. Each murine liver tissue was cut into several sections and frozen for triglyceride analysis.

### 2.7. Western Blot Analysis

Protein extracted from the liver was denatured and separated by mass in 10% Tris-glycine mini sodium dodecyl sulfate polyacrylamide gel electrophoresis (SDS-PAGE) and then transferred onto polyvinylidene difluoride (PVDF) membranes for protein blotting. The membranes were blocked with 5% dry milk for one hour at room temperature, then followed by incubation with primary antibodies for different targets detection at 4 °C overnight. Primary antibodies include protein kinase B (AKT; Cat. #4691, Cell Signaling Technology, Inc., Danvers, MA, USA), phosphorylated protein kinase B (pAKT; Cat. #4060, Cell Signaling Technologies), extracellular signal-regulated kinases (ERK1/2; Cat. #9102, Cell Signaling Technologies), phosphorylated extracellular signal-regulated kinases (pERK1/2; Cat. $4370, Cell Signaling Technologies), glycogen synthase kinase (GSK; Cat. #9315, Cell Signaling Technologies), phosphorylated glycogen synthase kinase 3 beta (pGSK-3β; Cat. #9322, Cell Signaling Technologies), and α-tubulin (Cat. #2125, Cell Signaling Technologies). The optimal dilutions of the antibodies were found to be 1:1000; α-tubulin was used as the internal control to normalize protein loading.

After overnight incubation, membranes were washed three times with 0.1% Tween 20-PBS, then incubated with goat anti-rabbit secondary antibodies at 1:1000 dilution for one hour at room temperature. Supersignal west femto maximum sensitivity substrate (Cat. # 34095, Thermo Fisher Scientific) was applied for detection. A half to five-second exposures were acquired on the Fujifilm Intelligent Dark Box II (Fujifilm, Mountain View, CA, USA).

### 2.8. Enzyme-Linked Immunosorbent Assay (ELISA)

Total protein in murine liver tissue was used to determine and compare the Chi3L1 levels in each study group. Hepatic levels of Chi3L1 protein were measured with a murine Chi3L1/YKL-40 ELISA kit (Cat# LS-F2800-1, Lifespan Biosciences, Inc., Seattle, WA, USA). The assay was performed according to the manufacturer’s instructions.

### 2.9. Hematoxylin & Eosin Staining

Paraffin-embedded murine liver tissue blocks were sectioned at 5 μm. Slides were baked at 60 °C, deparaffinized and hydrated through serial dilutions of ethanol (100%~90%~80%~70%) and stained with hematoxylin 30-s, 5 min dH_2_O, and 40-s eosin. Slides were then dehydrated through serial dilutions of ethanol (70%~80%~90%~100%) and xylene, then coverslipped with cytoseal.

### 2.10. Oil Red O (ORO) Staining

Frozen sections of murine liver were fixed in 10% neutral buffered formalin (NBF) for 10 min prior to staining and rinse. Slides were then treated with increasing levels of propylene glycol and stained with stock ORO solution (American MasterTech, Lodi, CA, USA) at room temperature for 20 min. The slides were then run through decreasing levels of propylene glycol and rinsed. The slides were stained with Harris hematoxylin and rinsed. Stained slides were observed and images were captured with an Olympus microscope (100×, 400× and 1000×, (Olympus, Waltham, MA 02453, USA) equipped with a digital camera. We used Image J v1.52 (NIH, Bethesda, MD, USA) to analyze the Oil Red O staining in each tissue slide, which indicated the amount of neutral lipids accumulation in the mouse liver.

### 2.11. Quantitative Liver Triglyceride Analysis

The biochemical assay was performed using a Triglyceride Colorimetric Assay Kit (No.10010303, Cayman Chemical, Ann Arbor, MI, USA). Glycerol and free fatty acids were released by lipase enzymatic hydrolysis of triglycerides in murine liver tissue homogenates, after centrifuging at 14,000 rpm for 15 min at 4 °C, the supernatant was collected for triglyceride measurement in a 96-well plate with a colorimetric readout at 540 nm.

### 2.12. Immunohistochemical Staining of Human Liver Biopsies for Chi3L1 Protein Expression

We conducted Chi3L1 immunohistochemistry staining on the paraffin-embedded human liver biopsy specimens. After the human liver tissue sections were deparaffinized, hydrated, and blocked with serum for one hour, they were incubated with affinity-purified goat polyclonal antibody prepared against purified human Chi3L1 protein (Catalog# AF2599, R & D Systems, Minneapolis, MN, USA) overnight at 4 °C and washed three times. Subsequently, the tissue sections were incubated with the secondary antibody which was included in the Vectastain Elite ABC HRP kit (Cat # PK-6105, Vector Laboratories, Burlingame, CA, 94010, USA). Positive staining indicated by a brown color was developed with DAB peroxidase substrate kit (Cat # SK-4100, Vector Laboratories). Hematoxylin was used as a counterstain.

### 2.13. Statistical Analysis

All the data were analyzed with Prism software. Results are expressed as the mean ± standard error of the mean (SEM). Either student’s t-test or one-way ANOVA was used for comparison. A *p* value of <0.05 was considered statistically significant.

## 3. Results

### 3.1. Knockout of the Chi3L1 Gene Enhanced Hepatic Insulin Signal Transduction

**Figure 1:**
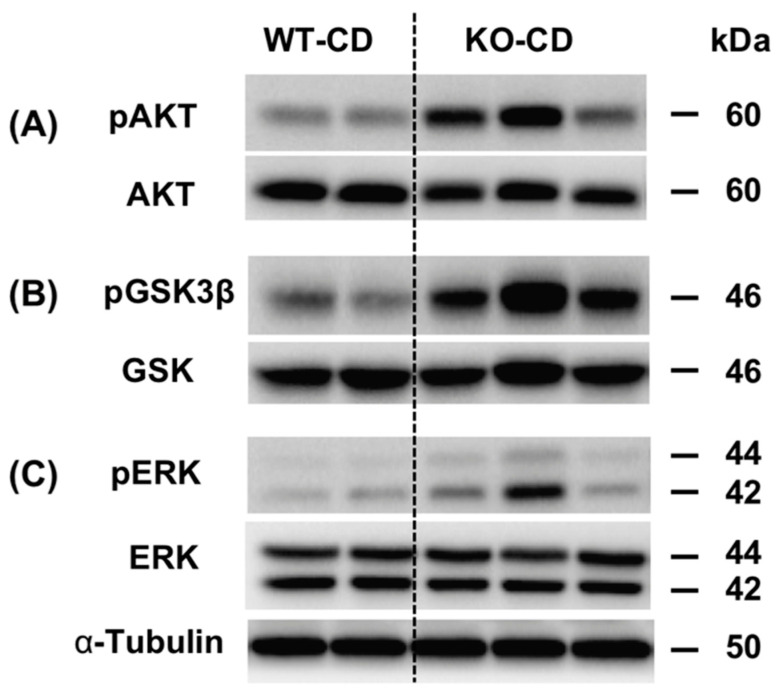
Hepatic insulin signal sensitivity is enhanced in Chi3L1 knockout mice fed with chow (CD). Western blot expression of (**A**) pAKT, (**B**) pGSK-3β, and (**C**) pERK proteins is enhanced in Chi3L1 knockout mouse liver 15 min after insulin injection (1 U/g body weight). WT: Wild type; CD: Chow diet; KO: knockout; pAKT: phosphorylated protein kinase B; AKT: Protein kinase B; pGSK-3β: phosphorylated glycogen synthase kinase 3 beta; GSK: glycogen synthase kinase; pERK: phosphorylated extracellular signal-regulated kinases; ERK: extracellular signal-regulated kinases.

We investigated the phosphorylation of AKT (pAKT) at serine 473 (Ser 473) and other downstream proteins such as pGSK3β and pERK, which play important roles in cell survival and proliferation, respectively after insulin stimulation. To determine if Chi3L1 expression influences hepatic insulin signal transduction, we performed Western blots to determine the levels of pAKT/AKT (Figure 1A), pGSK3β/GSK (Figure 1B) and pERK/ERK (Figure 1C) in CD fed WT and compared to CD fed KO mice. When the Chi3L1 gene was KO, increased expression of pAKT, pGSK-3β, and pERK was observed following i.p. insulin stimulation that enhanced transmission of the insulin signal in liver.

**Figure 2:**
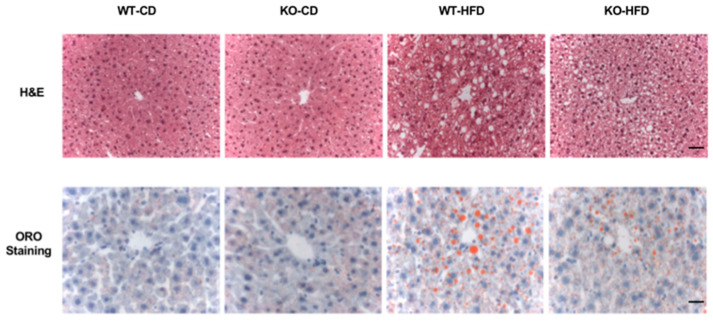
Lipid accumulation was reduced in Chi3L1 KO mice fed with HFD. Upper panel: representative Hematoxylin and eosin (H&E) staining, and demonstrate lipid accumulation in hepatocytes; Lower panel: representative Oil red O staining (ORO) and neutral fat accumulation in the murine liver (Bar = 20μm).

The H&E and ORO staining revealed that there was no significant difference in liver histology and lipid storage between WT and Chi3L1 KO mouse fed the CD. However, with a HFD, there was an increase in hepatic lipid deposition in WT mice along with neutral lipid accumulation (ORO staining). However, both were reduced in the KO mice fed the HFD (Figure 2).

### 3.2. High Fat Diet Increased Chi3L1 Gene and Protein Expression in the Liver of Wild Type C57BL/6 Mice

To investigate the effects of Chi3L1 expression on hepatic insulin resistance, we fed WT mice in group 1 with CD, while groups 2 to 5 were fed with HFD to induce hepatosteatosis (Table 1). All 40 mice in this experiment were euthanized after 16 weeks of feeding. The HFD significantly increased body weight in groups 2 to 5 compared to CD fed mice in group 1 (45.5 ± 1.4, 45.8 ± 0.6, 46.4 ± 1.2, and 44.3 ± 1.2 g vs. 33.0 ± 0.6, *p* < 0.001, Supplemental Figure S3). There were no significant differences in body weight between groups 2 to 5 mice fed with HFD.

**Figure 3:**
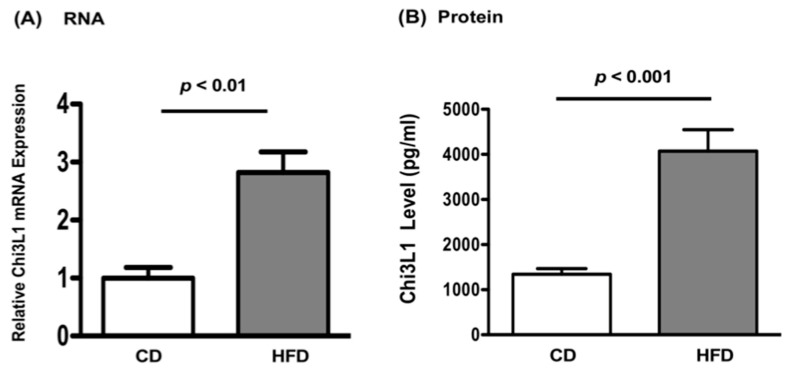
High fat diet (HFD) increased Chi3L1 gene and protein expression. (**A**) Relative Chi3L1 messenger RNA expression measured by Real time PCR. Data are expressed as Mean ± Standard Error of Mean (SEM), *p* < 0.01 vs. CD fed mice, N = 8. (**B**) Chi3L1 protein levels in mice livers measured by Chi3L1 ELISA. Results in each group are expressed as Mean ± Standard Error of Mean (SEM), *p* < 0.001 vs. CD fed mice, N = 8.

Real time PCR was used to evaluate Chi3L1 gene expression in the murine liver. When compared with CD fed mice, it was observed that HFD significantly increases Chi3L1 gene expression *p* < 0.01 (Figure 3A). The Chi3L1 protein levels were also measured by ELISA in cell lysates of the same liver samples. In comparison to CD fed mice, HFD significantly increased Chi3L1 protein levels in the liver as well (Figure 3B, 4073 ± 476.9 pg/mL vs. 1344 ± 123.9 pg/mL, *p* < 0.001).

### 3.3. Monoclonal Anti-Chi3L1 Antibodies’ Administration Improves Hepatic Insulin Signaling in HFD Fed Mice

#### 3.3.1. Effects of FRG on the Hepatic Insulin Signaling Cascade

**Figure 4:**
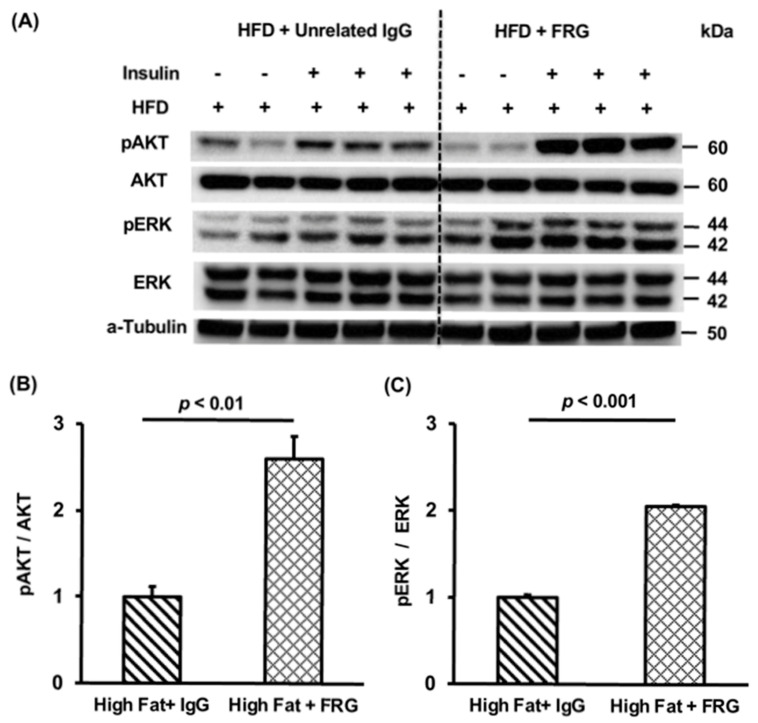
Effects of monoclonal anti-Chi3L1 antibody- FRG treatment on hepatic insulin signal transduction. (**A**) Hepatic insulin signaling in the murine liver measured by Western blot. High fat diet (HFD) fed mice were divided into two groups, five mice in each group were treated with either unrelated IgG or FRG (200 μg i.p twice/week) for 16 weeks. Three mice in each group were stimulated by insulin injection (1U/gram body weight) while the remainder were not injected with insulin before sacrifice. (**B**,**C**) Quantification by densitometry of pAKT and pERK expression in the mouse liver by Western blot analysis. *p* < 0.01, *p* < 0.001 vs. high fat diet + IgG treated mice, N = 3.

The FRG is one of three anti-Chi3L1 mAbs that were administered to mice for 16 weeks. Control mice received unrelated IgG at the same dose and frequency. We evaluated the effects of FRG administration on hepatic insulin signaling as well as lipid accumulation in the liver. The HFD induced insulin resistance following stimulation (Figure 4A). It was observed that FRG administered twice a week significantly increased pAKT/AKT (Figure 4B, *p* < 0.01 vs. unrelated IgG treated control) and pERK/ERK expression (Figure 4C, *p* < 0.001).

#### 3.3.2. Effects of Anti-Chi3L1 mAbs (CH568 and CHXI3B6) on Hepatic Insulin Resistance

**Figure 5:**
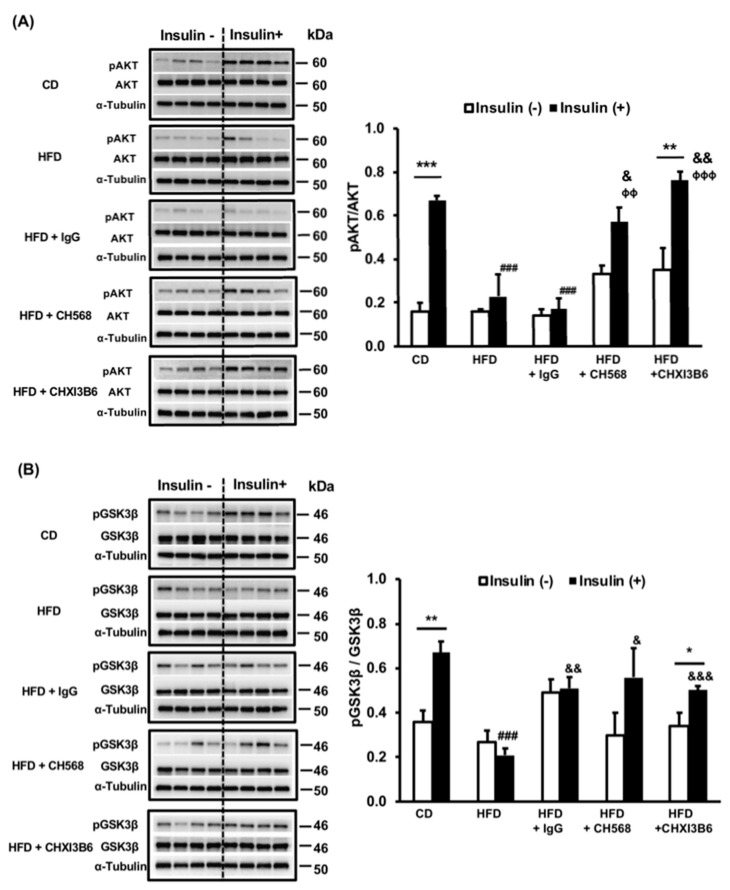
Effect of anti-Chi3L1 mAbs on hepatic insulin signaling. (**A**) The expression of pAKT and AKT in the murine liver. The lower right panel showed the quantification by densitometry of Western blot results. ** *p* < 0.01, *** *p* < 0.001 vs. insulin (−) in the same group; ### *p* < 0.001 vs. CD insulin (+); & *p* < 0.05, && *p* < 0.01 vs. HFD insulin (+) ᶲᶲ *p* < 0.01, ᶲᶲᶲ *p* < 0.001 vs. HFD + IgG insulin (+), N = 4. (**B**) The expression of pGSK-3β and GSK in the murine liver. In the upper panel, the Western blot results for pGSK-3β and GSK protein expression are shown. The lower panel shows the quantification of Western blot data. * *p* < 0.05, ** *p* < 0.01 vs. insulin (−) in the same group; ### *p* < 0.001 vs. CD insulin (+); & *p* < 0.05, && *p* < 0.01, &&& *p* < 0.001 vs. HFD insulin (+), N = 4.

To further confirm the observations of FRG above, we expanded the experiments to include 40 WT C57BL/6 mice (Table 1). Two additional anti-Chi3L1 mAbs (CH568 and CHXI3B6) were used in this experiment and revealed similar results to the FRG treated mice with respect to enhanced pAKT and pGSK3β generation after administration (Figure 5). However, there was no difference in pERK formation (Supplemental Figure S4). Thus, altering Chi3L1 function by CH568 and CHXI3B6 administration twice a week also reduced hepatic insulin resistance.

### 3.4. The Anti-Chi3L1 mAb Treatment Significantly Reduced Lipid Accumulation in HFD Fed Murine Liver

**Figure 6:**
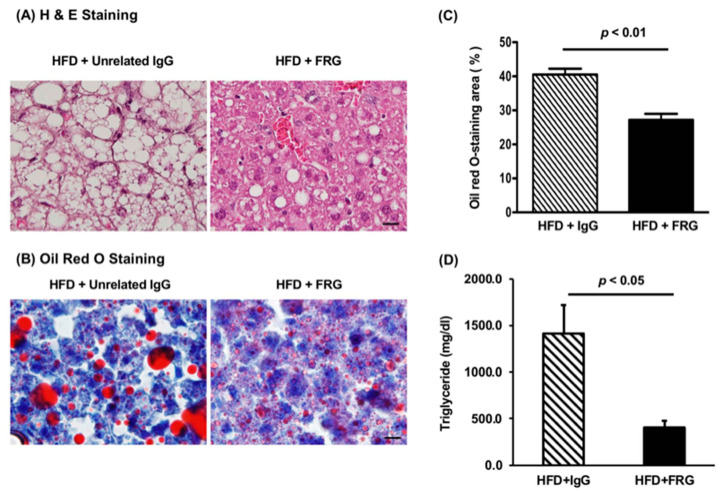
Monoclonal anti-Chi3L1 (FRG) antibody treatment reduced lipid accumulation in the mouse liver. (**A**) Representative H&E staining; (**B**) Representative Oil Red O (ORO) staining; (**C**) Quantitative analysis of ORO staining. All mice were treated with HFD for 16 weeks. The original magnification is 1000 x. *p* < 0.01 vs. HFD + IgG, N = 5. (**D**) Triglyceride content in the mouse liver tissue. Results were expressed as Mean ± Standard Error of Mean (SEM), *p* < 0.05 vs. HFD + IgG, N = 5 (Bar = 20 μm).

In order to determine if the anti-Chi3L1 mAb administration could also affect lipid accumulation, we examined H & E stained slides (Figure 6A) and evaluated neutral fat accumulation by ORO staining (Figure 6B,C) as well as measured triglyceride levels (Figure 6D). The results revealed that FRG treatment substantially reduced the size of the fat droplets in the cytoplasm of hepatocytes (Figure 6A). The ORO staining (Figure 6B) and quantification analysis demonstrated that FRG administration significantly reduced neutral lipid accumulation (Figure 6C, *p* < 0.01). Triglyceride levels in FRG treated mice were also significantly lower than that in the unrelated IgG treated control group (Figure 6D, *p* < 0.05).

**Figure 7:**
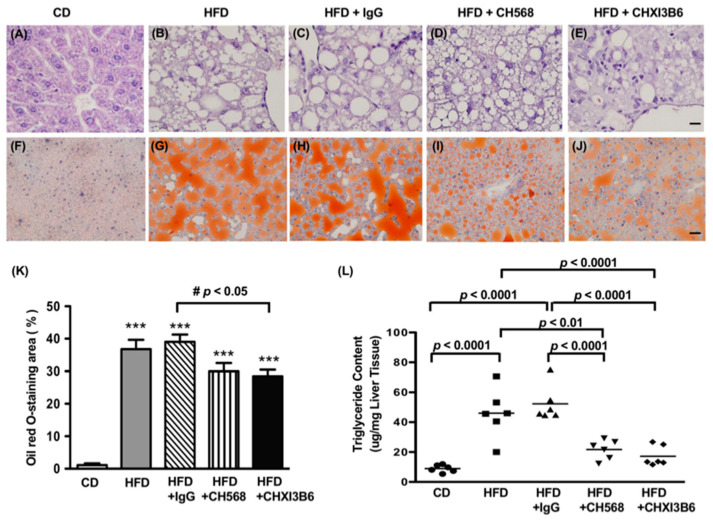
Monoclonal anti-Chi3L1 antibodies treatment reduced lipid accumulation in HFD fed WT C57BL/6 liver. (**A**–**E**) Representative H&E. Original magnification is 1000 x (Bar = 20 μm). (**F**–**J**) Representative Oil red O (ORO) staining (Bar = 20 μm). (**K**) Analysis of ORO staining for neutral fat accumulation in each group. The x-axis represents group information for each experimental setting, while the y-axis represents the percentile of the ORO area in the murine liver. *** *p* < 0.0001 vs. CD fed mice, # *p* < 0.05 vs. HFD+ unrelated IgG treated mice, N = 8. (**L**) Triglyceride content in the mouse liver. Results (horizontal bars) in each group were expressed as Mean ± Standard Error of Mean (SEM). Each symbol in the figure represents one animal in each group, N = 6.

The WT C57BL/6 mice fed the HFD for 16 weeks, resulted in severe steatosis (Figure 7A). The CD fed mice exhibited normal liver structure with healthy hepatocytes, while ballooned hepatocytes along with infiltration of polymorpho-leukocytes were observed in the HFD fed mice (Figure 7B–E). The cytoplasm of hepatocytes was filled with large lipid droplets in HFD fed mice (Figure 7B,C). There was less extensive lipid vacuoles accumulation in the cytoplasm of hepatocytes in anti-Chi3L1 (CH568 and CHXI3B6) mAbs treated mice (Figure 7D,E).

Representative ORO staining images are shown in Figure 7F–J and revealed that HFD produced increased neutral fat accumulation in the cytoplasm of hepatocytes (Figure 7G–J). The size of lipid droplets decreased in anti-Chi3L1 mAb treated mice as represented in groups 4 and 5 (Figure 7I,J) in Table 1. In comparison with the CD, HFD significantly increased neutral lipid accumulation in the murine liver (*p* < 0.0001 vs. CD, Figure 7K) which was reduced by anti-Chi3L1 mAb treatment.

The role of these mAbs in reducing lipid deposition in hepatocytes were further analyzed by total hepatic triglyceride measurements. As shown in Figure 7L, compared to CD fed mice, HFD fed mice had a significantly higher total triglyceride content in the liver. In comparison with the HFD fed mice in group 2, anti-Chi3L1 mAb administration significantly reduced the triglyceride levels of the HFD fed mice in groups 4 and 5 (21.7, 25.2 vs. 46 μg/mg tissue, *p* < 0.01, *p* < 0.0001 vs. HFD, Figure 7L).

Chi3L1 Is Highly Expressed in Human Liver with NAFLD/NASH Pathology

**Figure 8:**
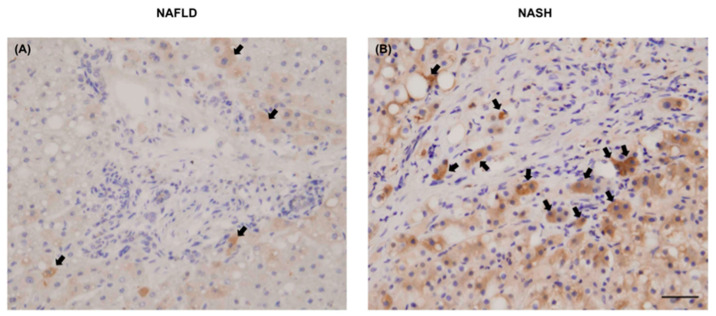
Representative Chi3L1 IHC staining for expression in human liver biopsy specimens. (**A**) NAFLD liver tissue. (**B**) NASH liver disease. Arrows indicated specific anti-Chi3L1 antibody stained cells including hepatocytes. Original magnification is 400× (Bar = 20 μm).

To determine if Chi3L1 was expressed in human diseased liver, we obtained 20 archival liver biopsy specimens and examined Chi3L1 cellular expression by performing IHC (Figure 8). Both NAFLD and NASH derived liver biopsies (N = 5 each) showed positive staining of macrophages, inflammatory cells and hepatocytes. However, the extent and intensity of positive staining was far greater in NASH than NAFLD derived biopsy specimens and further quantitative studies will be required. Indeed, a complete phenotypic analysis of the type and characteristics of the inflammatory cells infiltrating the liver by immunohistochemistry will be of interest in a larger cohort of NASH and NAFLD patients in the context of increased Chi3L1 expression and a focus in future studies. Representative images revealed that Chi3L1 protein was expressed at low levels in hepatocytes of patients with NAFLD (Figure 8A). They were particularly prominent in portal area of liver. Furthermore, expression of Chi3L1 protein was abundantly expressed in hepatocytes (black arrows) with a pathologic diagnosis of NASH (Figure 8B). Positively stained cells were primarily localized to portal areas as well.

## 4. Discussion

There are several animal models that develop a NAFLD/NASH-like disease that involves inflammation and steatosis [36,37,38,39,40]. In this context, we have evaluated the role of Chi3L1 in the development of insulin resistance by comparing insulin signaling in a Chi3L1 KO murine model to wild type (WT) mice C57BL/6 fed a CD. The results indicate that insulin signal transduction is improved in Chi3L1 gene KO mice compared to WT animals fed the same diet and suggest that reducing Chi3L1 hepatic expression or function could improve insulin resistance in the liver. The WT C57BL/6 develop NAFLD/NASH like pathology on HFD in association with enhanced Chi3L1 gene expression levels which allowed us to access the role of this gene in the pathogenesis of insulin resistance. It will be important to examine the role of stellate cells that also have upregulated Chi3L1 protein expression on hepatocyte injury, insulin resistance and fibrosis as described [41,42].

We focused on the interaction of Chi3L1 protein with the hepatic insulin signaling cascade as measured by Western blot analysis to clarify the activation of these cellular proteins critical for transmitting the insulin signal and subsequently involved in hepatocyte survival and proliferation. Thus, the expression of pAKT, and downstream signaling molecules pGSK-3β and pERK in the liver after in vivo insulin stimulation [33] were quantified.

Without insulin stimulation, pAKT, pGSK-3β and pERK expression in the murine liver is very low (Figure 5). However, 15 min after direct i.p. insulin stimulation, a significant increase in pAKT and pGSK-3β expression was observed (Figure 5A,B). In contrast, HFD significantly blunted the insulin stimulated activation of pAKT and pGSK-3β (*p* < 0.001 and *p* < 0.01, respectively, vs. the CD insulin stimulated group). When comparing expression levels of pAKT/AKT, pGSK/GSK and pERK/ERK with the HFD fed mice represented in group 2, both murine anti-Chi3L1 mAbs treated HFD fed mice in groups 4 and 5 demonstrated partially restored insulin sensitivity (*p* < 0.05, *p* < 0.01 and *p* < 0.001 vs. the HFD) (Figure 5A,B) suggesting that the function of Chi3L1 protein had been altered. In contrast, unrelated IgG injection in group 3 had no effect on hepatic insulin sensitivity.

Insulin is a key anabolic hormone involved in carbohydrate and fat metabolism [43,44] and the liver is one of the most insulin-responsive organs [44,45]. Hepatic insulin resistance plays an important role in the development of NASH [1,26,27]. Similar to other studies, HFD-fed C57BL/6 mice gained excessive weight and developed NAFLD/NASH histology within 16 weeks of HFD feeding [36,37,38,39,40,46]. The development of this phenotype was associated with upregulation of the Chi3L1 gene at both the RNA and protein levels and supports its role in modulating insulin sensitivity of the liver. It will be essential to investigate other signaling molecules like the insulin receptor (IR) or insulin receptor substrate 1 and 2 (IRS-1 and IRS-2) and their tyrosyl phosphorylation [8,9] as well as downstream insulin responsive gene expression to further characterize the insulin resistance phenotype [8,9].

There are two major pathways involved in the hepatic insulin signaling cascade and the PI3K/AKT pathway is responsible for most metabolic effects of insulin [43,47,48,49]. The pAKT, and pGSK-3β are involved in the cell survival pathway and pERK is important in mitogenesis [43,50]. Our results indicate that when Chi3L1 gene was knocked out, pAKT, pGSK-3β, and pERK expression were substantially enhanced following in vivo insulin stimulation. In addition, comparing with the WT mice fed with HFD to the KO mice, it was observed that lipid accumulation in the liver was reduced as well. Moreover, excessive Chi3L1 gene expression induced by HFD markedly blunted hepatic insulin signaling as measured by reduced pAKT, pGSK-3β, and pERK levels. These findings suggest that Chi3L1 gene upregulation may play a role in the development of hepatic insulin resistance associated with inflammation and lipid deposition. Based on previous observations, there are four putative cellular signaling pathways interacting with Chi3L1: (1) binding to IL-13 receptor α2 (IL-13Rα2), which activates the phosphoinositide-3 kinase (PI3K) cascade; (2) stimulation of the mitogen activated protein kinases (MAPK) pathway which involves ERK; (3) the chitin-binding motif of Chi3L1 appears to stimulate the AKT signaling pathway; and (4) recombinant mouse Chi3L1 increased pAKT expression in cultured mouse macrophages derived from Chi3L1^−/−^ mouse livers [16,51,52,53]. However, the role of Chi3L1 on insulin stimulated activation of these molecules have not been investigated.

The Chi3L1 gene is also involved in mediating inflammation, tissue remodeling, fat accumulation and cancer [12,19,20,21,42]. Moreover, investigations have suggested that Chi3L1 serum levels correlate with tissue injury and the degree of hepatic fibrosis [52]. Overexpression induced by HFD may contribute to the lipid deposition and targeting Chi3L1 function or reducing its level may improve insulin resistance and reduce steatohepatitis. This hypothesis is supported by interfering with Chi3L1 protein function through administration of anti-Chi3L1 mAbs which improved hepatic insulin sensitivity and reduced lipid accumulation in hepatocytes.

These studies may have biologic relevance to human disease. Although previous RNA sequencing data from the Illumina Human Body Map 2.0 (http://genomicdbdemo.bxgenomics.com) showed that the Chi3L1 gene was highly expressed in human liver [22], it remained unknown if Chi3L1 is expressed in human hepatocytes in the context of developing a NAFLD/NASH phenotype. We observed localization of Chi3L1 expressing cells in the portal areas of NAFLD/NASH liver. The IHC staining images revealed that it was abundantly expressed not only in infiltrated inflammatory cells, but in hepatocytes as well.

Therefore, Chi3L1 gene is highly expressed in both human and murine liver with NAFLD/NASH histology and all three anti-Chi3L1 mAb proteins, to variable degrees, inhibit its function in vivo and substantially improve hepatic insulin resistance following 16 weeks of anti-Chi3L1 mAb treatment. Indeed, western blot analysis of key proteins (pAKT, pGSK-3β, and pERK) in the insulin signaling cascade revealed that all three anti-Chi3L1 mAbs (FRG, CH568 and CHXI3B6) were effective in improving insulin resistance and reducing lipid accumulation in the murine liver. Histological lipid grading together with Oil Red O staining results, and measurement of triglyceride levels suggest that altering Chi3L1 protein function with specific targeted agents to Chi3L1 may be beneficial in reducing hepatic steatosis as well.

In conclusion, it was demonstrated that KO of Chi3L1 gene enhanced hepatic insulin signal transduction in CD fed mice and limit lipid accumulation induced by HFD. The HFD significantly increased Chi3L1 expression levels in normal murine liver and it was highly expressed in human liver with NAFLD/NASH histology. Monoclonal antibodies targeting Chi3L1 protein function may significantly improve insulin sensitivity and decrease lipid accumulation and may alter the progression of this serious liver disease.

## Figures and Tables

**Table 1:** Experimental feeding regimen.

Groups	N	Diet	Treatment	Insulin Stimulation
Group 1	8	Chow Diet	None	+ (4 mice)
				− (4 mice)
Group 2	8	High Fat Diet	None	+ (4 mice)
				− (4 mice)
Group 3	8	High Fat Diet	Unrelated IgG 200 µg/mouse, twice a week	+ (4 mice)
				− (4 mice)
Group 4	8	High Fat Diet	Chi3L1 IgG Ab. (CH568), 200 µg/mouse, twice a week	+ (4 mice)
				− (4 mice)
Group 5	8	High Fat Diet	Chi3L1 IgG Ab. (CHXI3B6), 200 µg/mouse, twice a week	+ (4 mice)
				− (4 mice)

All the mice were fed with either CD or HFD and treated with or without monoclonal anti-Chi3L1 IgG antibodies for 16 weeks. There are eight wild type mice in each group and half of the mice in each group were injected with insulin 15 min before dissection and removal of the liver. Group 1 mice fed with CD served as control; Group 2 mice were fed with HFD; Group 3 mice were fed with HFD and treated with unrelated IgG mAb; Group 4 and 5 mice were fed with HFD and treated with monoclonal anti-Chi3L1 IgG mAbs CH568 or CHXI3B6, respectively; +: with insulin stimulation; −: without Insulin stimulation.

## Data Availability

The data presented in this study are available on request from the corresponding author.

## Supplementary Materials

The following are available online at https://www.mdpi.com/2073-4409/10/2/201/s1, Figure S1: Characterization of monoclonal anti-Chi3L1 antibodies; Figure S2: Time course of insulin stimulation of murine liver; Figure S3: Effect of anti-Chi3L1 mAbs on the phosphorylation of extracellular signal-regulated kinases (ERK) in the mouse liver; Figure S4: High fat diet significantly increased mouse body weight.
