# Supplementary material for: Role of Chitinase 3-Like 1 Protein in the Pathogenesis of Hepatic Insulin Resistance in Nonalcoholic Fatty Liver Disease
Source: Cells. 2021 Jan 20;10(2):201. doi: 10.3390/cells10020201 (PMC7909438; doi:10.3390/cells10020201)
Supplement: Supplementary file 1 [file cells-10-00201-s001.pdf]

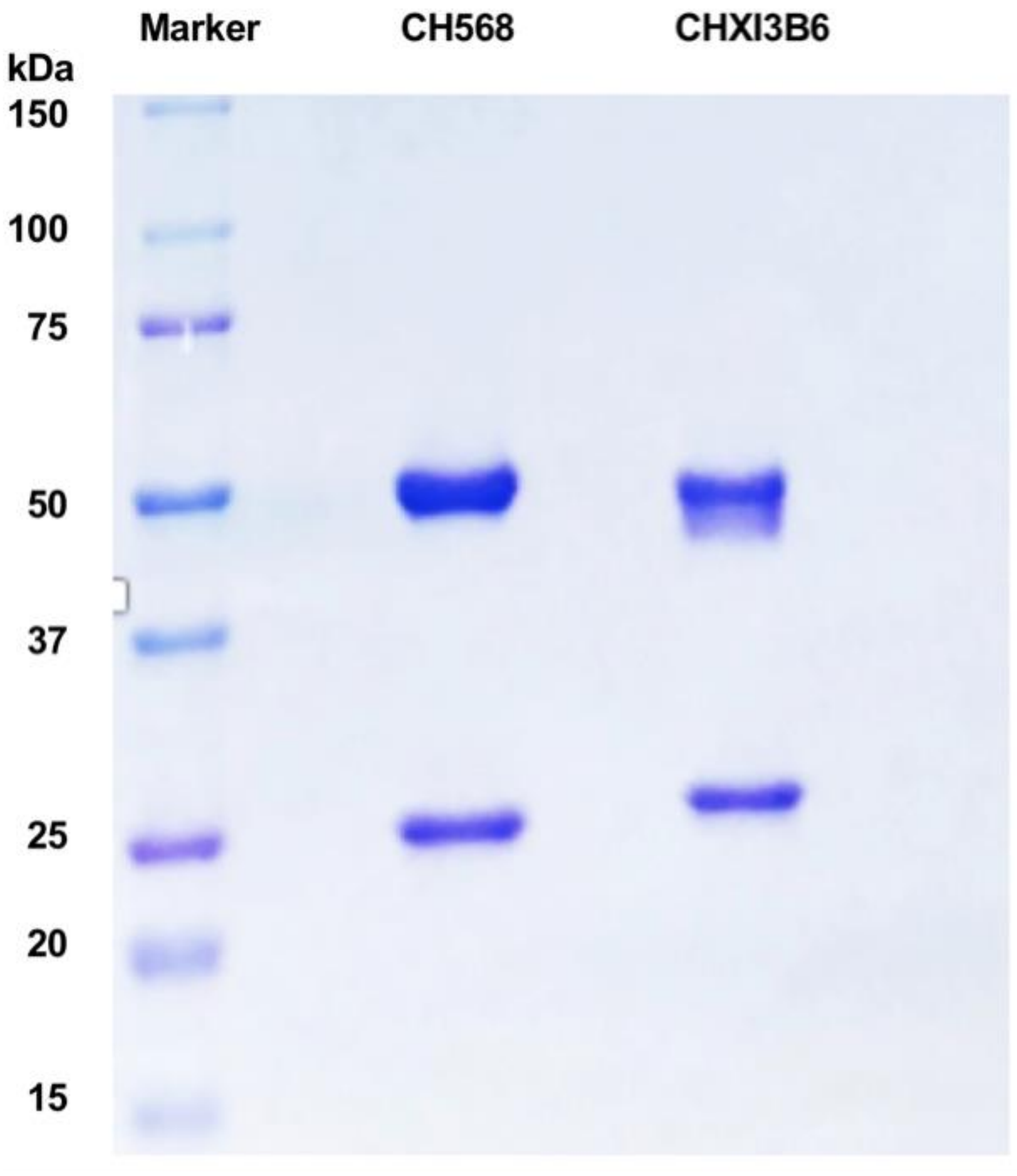

Supplemental Figure 1. Characterization of monoclonal anti-Chi3L1 antibodies. Purified antibodies were denatured and separated on a precast 4-20% SDS-PAGE gel. Two single bands at 50 kD (heavy chain) and 25 kD (light chain) in each lane are shown for each anti-Chi3L1mAb, CH568 and CHXI3B6.

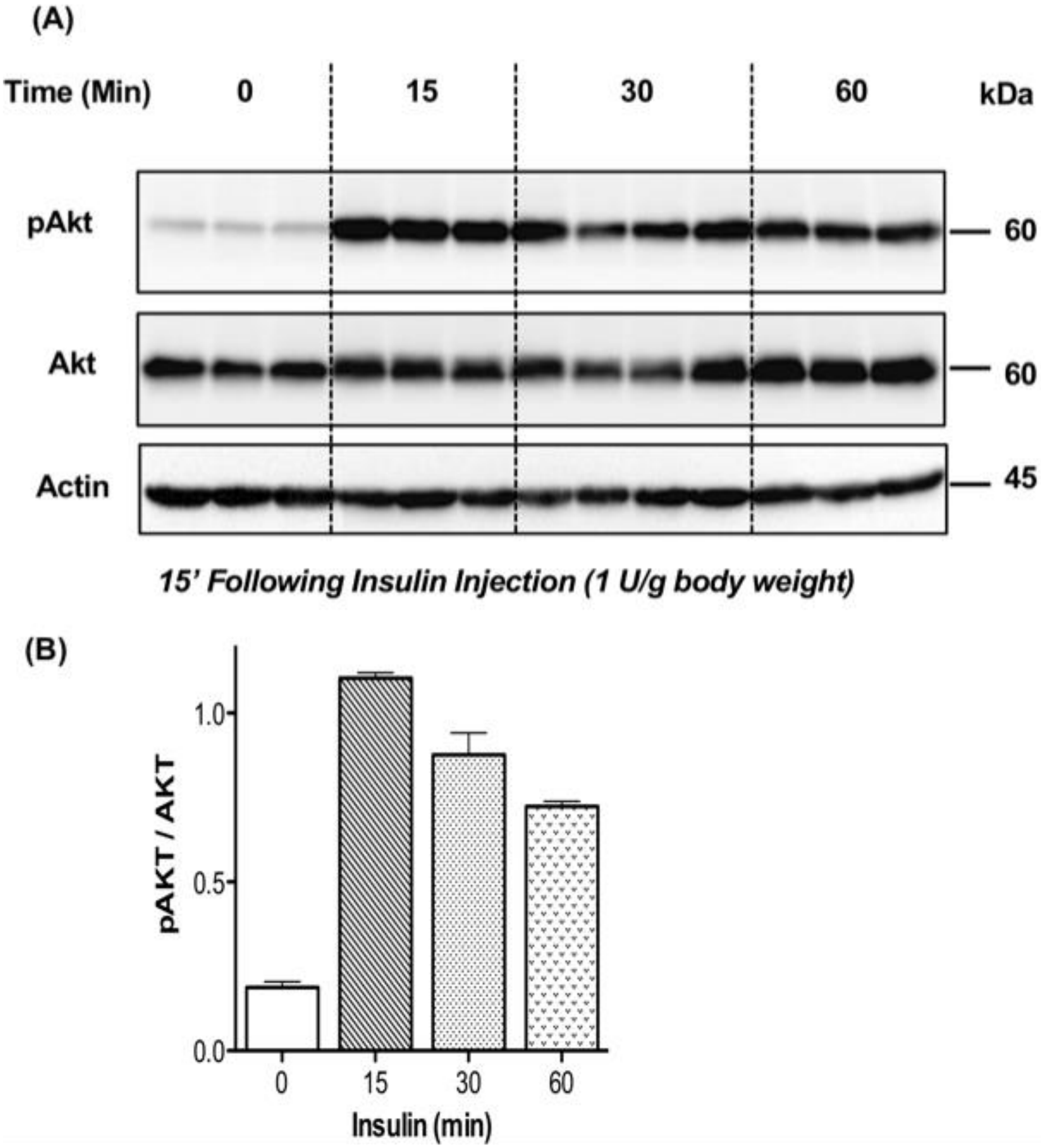

Supplemental Figure 2. Time course of insulin stimulation of murine liver. **(A)** pAKT expression measured by Western blot. **(B)** Quantification of the Western blot results.

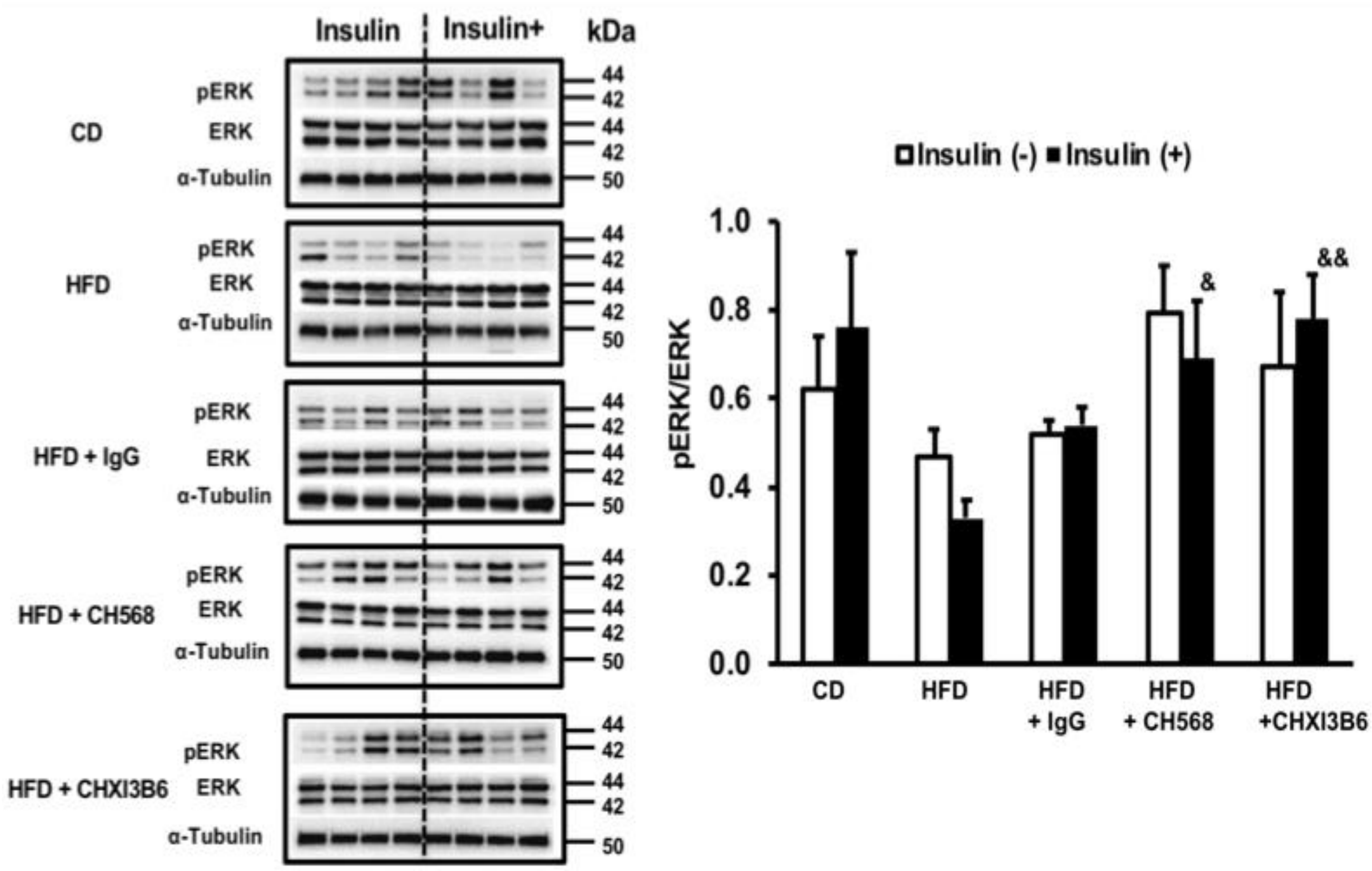

Supplemental Figure 3. Effect of anti-Chi3L1 mAbs on the phosphorylation of extracellular signal-regulated kinases (ERK) in the mouse liver. The expression of α-Tubulin was used as a loading control for comparison. There was no effect of these two anti-Chi3L1 mAb treatment on generation of pERK by insulin stimulation.

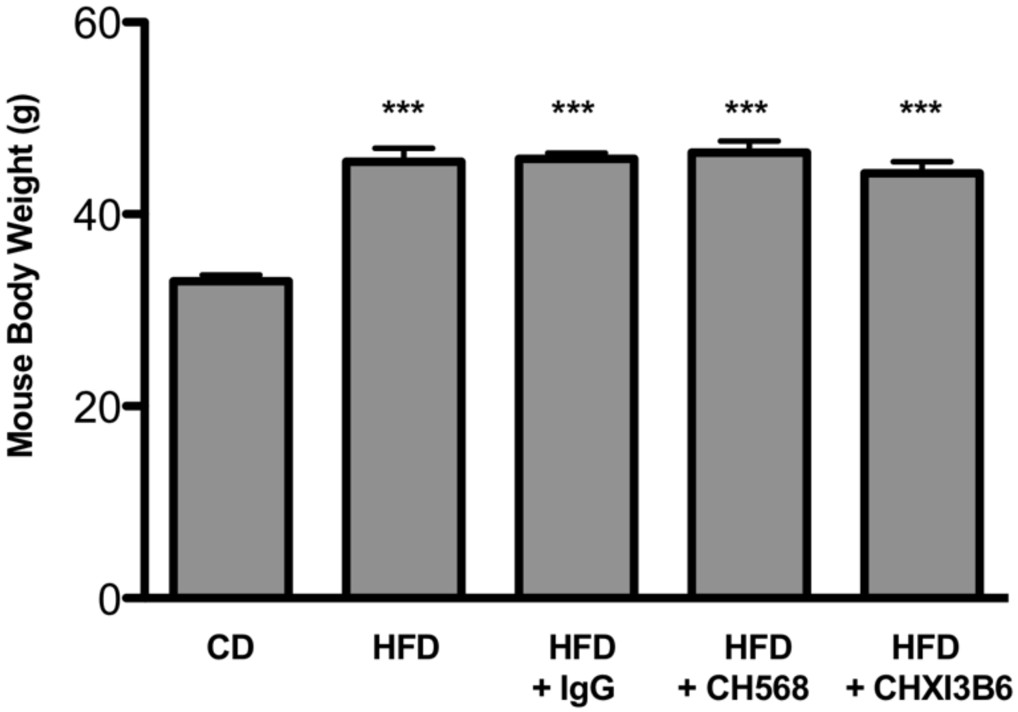

Supplemental Figure 4. High fat diet significantly increased mouse body weight. The y-axis is the average body weight measured across eight mice in each group. Data are expressed as Mean ± Standard Error of Mean (SEM), *** $p<0.0001$ *vs* CD, N=8.
